# Identification of necroptosis subtypes and development of necroptosis-related risk score model for in ovarian cancer

**DOI:** 10.3389/fgene.2022.1043870

**Published:** 2022-12-08

**Authors:** Chen Ji, Yue He, Yan Wang

**Affiliations:** Department of Gynecological Oncology, Beijing Obstetrics and Gynecology Hospital, Beijing Maternal and Child Health Care Hospital, Capital Medical University, Beijing, China

**Keywords:** necroptosis, ovarian cancer, classification of subtypes, risk model, prognosis, immune infiltration

## Abstract

**Background:** ith the ongoing development of targeted therapy, non-apoptotic cell death, including necroptosis, has become a popular topic in the field of prevention and treatment. The purpose of this study was to explore the effect of necroptosis-related genes (NRGs) on the classification of ovarian cancer (OV) subtypes and to develop a necroptosis-related risk score (NRRS) classification system.

**Methods:** 74 NRGs were obtained from the published studies, and univariate COX regression analysis was carried out between them and OV survival. Consensus clustering analysis was performed on OV samples according to the expression of NRGs related to prognosis. Furthermore, the NRRS model was developed by combining Weighted Gene Co-Expression Network Analysis (WGCNA) with least absolute shrinkage and selection operator (Lasso)-penalized Cox regression and multivariate Cox regression analysis. And the decision tree model was constructed based on the principle of random forest screening factors principle.

**Results:** According to the post-related NRGs, OV was divided into two necroptosis subtypes. Compared with Cluster 1 (C1), the overall survival (OS) of Cluster 2 (C2) was significantly shorter, stromal score and immune score, the infiltration level of tumor associated immune cells and the expression of 20 immune checkpoints were significantly higher. WGCNA identified the blue module most related to necroptosis subtype, and 12 genes in the module were used to construct NRRS. NRRS was an independent prognostic variable of OV. The OS of samples with lower NRRS was significantly longer, and tumor mutation burden and homologous recombination defect were more obvious.

**Conclusion:** This study showed that necroptosis plays an important role in the classification, prognosis, immune infiltration and biological characteristics of OV subtypes. The evaluation of tumor necroptosis may provide a new perspective for OV treatment.

## Introduction

Ovarian cancer (OV) is the deadliest cancer in the female reproductive tract (Whelan et al., 2022). Worldwide, 313,959 new diagnosed ovarian cancer and 207,252 succumb to this disease in 2020 (Sung et al., 2021). OV is usually confined to the peritoneal cavity throughout its course, with occasional distant metastases. Due to vague and non-specific signs and symptoms, and limited screening methods, the initial diagnosis is usually delayed until extensive intraperitoneal diffusion occurs through the adjacent peritoneal surface, ascites and rich lymphatic vessels (Achimas-Cadariu et al., 2022). According to statistics, about 3/4 of OV patients are diagnosed with advanced stage, and the prognosis is disappointing (Zhang et al., 2022). OV also faces a large number of unsolved problems such as difficult choice of treatment strategies and high recurrence rate (Rakina et al., 2022). Surgical treatment is currently recognized as the best method for the treatment of ovarian cancer, and platinum-paclitaxel chemotherapy as adjuvant therapy can significantly improve the effectiveness of ovarian cancer treatment. (van Stein et al., 2021; Wood and Ledermann, 2022). However, OV a highly heterogeneous at the molecular level, therefore, molecular targeted therapy is considered as a less toxic but more effective treatment in OV (Guan and Lu, 2018). For example, the combined application of PARP inhibitors, anti-VEGF monoclonal antibody and ICI has become a research hotspot (Revythis et al., 2022). Better understanding the biological characteristics and molecular heterogeneity of OV in order to formulate or improve treatment strategies and improve quality of life is an urgent demand (Zhang et al., 2022).

Apoptotic cell death plays an important role in OV (Hou et al., 2019). Ongoing development of targeted therapy allows non-apoptotic cell death to become popular in the field of prevention and treatment, including ferroptosis, alkaliptosis, autophagy, necroptosis, pyroptosis, immunogenic cell death as well as other cell death modes (Chen et al., 2022a). Necroptosis is a form of programmed necrosis, which differs from apoptosis as caspases activation is not involved in its progression. Instead, it is mediated by external signals, which trigger the activation of Mixed-Lineage Kinase Domain-Like (MLKL) signaling cascade, Receptor Interacting Protein 1 (RIP1), RIP3 (Liu et al., 2022). It is characterized by mitochondrial changes and plasma membrane permeability, resulting in the release of cytoplasmic contents into extracellular space and inflammation (Beretta and Zaffaroni, 2022). Preclinical and clinical evidence show that it is the outstanding pro-inflammatory characteristics of necroptosis that contribute to the correlation between necroptosis and cancer pathophysiology (Pasparakis and Vandenabeele, 2015). Necroptosis is regulated by molecular mechanism. Targeting necroptosis has shown substantial potential in tumor treatment with small molecules may have the advantage of bypassing the mechanism of apoptosis resistance (Wu et al., 2022). There is growing evidence that necrosis plays a key role in the development and progression of a wide range of diseases, including neurodegenerative diseases, ischemic cardiovascular disease and cancer metastases (Gong et al., 2019; Chen et al., 2022b). In addition, necrosis has a dual role in promoting and inhibiting tumor growth in a variety of tumor types (Seifert et al., 2016; Strilic et al., 2016; Qin et al., 2019). Therefore, from this point of view, the key molecular insights on necroptosis provide a prospect for targeted therapy. The key molecules of necroptosis have been poorly studied in OV.

In the current study, based on the cluster analysis of transcriptional profiles of necroptosis-related genes, we identified the necroptosis subtypes of OV, and described the clinical and molecular characteristics, immune characteristic and association with immunotherapy response. A necroptosis-related risk score (NRRS) model was developed by Weighted Gene Co-Expression Network Analysis (WGCNA) and least absolute shrinkage and selection operator (LASSO) Cox regression analysis, and a clinical decision tree model and nomogram were established to improve the risk stratification of survival in OV patients.

## Materials and methods

### Extraction and preprocessing of OV cohort data

The RNA sequencing (RNA-seq), somatic mutation, copy number alterations (CNAs) data and clinical follow-up information of OV were found and downloaded in The Cancer Genome Atlas (TCGA, https://portal.gdc.cancer.gov/) database. In International Cancer Genome Consortium (ICGC, https://dcc.icgc.org/), the samples with detailed RNA-seq and clinical survival data were also included in the analysis. Another OV cohorts (GSE26193, GSE30161, GSE63885, GSE9891) were collected from the Gene Expression Omnibus (GEO, https://www.ncbi.nlm.nih.gov/geo/) database. The clinical features were showed in Table 1.

**TABLE 1:** The clinical features of datasets

	TCGA	ICGC	GSE26193	GSE30161	GSE63885	GSE9891
	(N=373)	(N=93)	(N=107)	(N=58)	(N=70)	(N=276)
OS						
0	143 (38.3%)	19 (20.4%)	31 (29.0%)	22 (37.9%)	4 (5.7%)	163 (59.1%)
1	230 (61.7%)	74 (79.6%)	76 (71.0%)	36 (62.1%)	66 (94.3%)	113 (40.9%)
Age						
Mean (SD)	59.6 (11.4)			62.6 (10.6)		59.6 (10.5)
Median [Min, Max]	59.0 [30.0, 87.0]			62.0 [38.0, 85.0]		59.0 [22.0, 80.0]
Stage						
I	1 (0.3%)		21 (19.6%)			24 (8.7%)
II	21 (5.6%)		10 (9.3%)		1 (1.4%)	17 (6.2%)
III	291 (78.0%)	79 (84.9%)	59 (55.1%)	53 (91.4%)	59 (84.3%)	212 (76.8%)
IV	57 (15.3%)	14 (15.1%)	17 (15.9%)	5 (8.6%)	10 (14.3%)	22 (8.0%)
Missing	3 (0.8%)					1 (0.4%)
Grade						
G1	1 (0.3%)					
G2	42 (11.3%)			25 (43.1%)	8 (11.4%)	
G3	319 (85.5%)			33 (56.9%)	44 (62.9%)	
G4	1 (0.3%)				18 (25.7%)	
Missing	10 (2.7%)					

### Consensus clustering analysis was performed on OV samples by obtaining necroptosis related genes

A study by Xin et al. (2022) gave 74 necroptosis-related genes (NRG). Univariate COX regression analysis was carried out to screen NRGs related to prognosis. R package “ConsensusClusterPlus” root conducted the unsupervised hierarchical clustering of OV according to expression of prognosis-related NRGs. Euclidean distance and “pam” were utilized to compute the similarity distance between samples, with 500 iterations and 80% resampling rate, ranging from 2 to 10. The final optimal clustering number, was defined by the cumulative distribution function (CDF) curve and delta area, showed high consistency within the cluster, low variation coefficient without significant change in the area under the CDF curve.

### Detection of tumor mutation

The “maftools” package (Mayakonda et al., 2018) was employed to analyze and visualize the single nucleotide variation (SNV) data processed by mutect2 in TCGA. Firstly, the genes with mutation frequency >3 in the sample were screened, and the statistical differences of high frequency mutation genes between subgroups were analyzed by fisher test, and the mutations of 20 genes with the highest mutation rate in different subgroups were shown by waterfall map. For number of segments and tumor mutation burden (TMB), fraction altered, homologous recombination defects, between subgroups, Wilcoxon test was used to compare.

### Assessment of tumor immune microenvironment

The proportion of immune cells in tumor microenvironment (TME) was estimated by marker genes expressions-based microenvironment cell population (MCP) counter (Becht et al., 2016) and single sample gene set enrichment analysis (ssGSEA), and the results were expressed as enrichment scores. ESTIMATE (Estimation of STromal and Immune cells in MAlignant Tumours using Expression data) (Yoshihara et al., 2013) was used to calculate the stromal score and immune score and ESTIMATE score of the sample to quantify the overall level of TME matrix and infiltrating immune components.

### Prediction of immunotherapy response

Immune checkpoint expression from HisgAtals and TIDE score from tumor immune dysfunction and exclusion (TIDE, http://tide.dfci.harvard.edu) (Jiang et al., 2018) were used to evaluate the immune checkpoint inhibitors treatment response between different OV subgroups. Different TIDE scores represent different sensitivities to immunotherapy, and low TIDE score is considered to be responsive to immunotherapy.

### Weighted gene co-expression network analysis

To identify the key modules that are highly related to the OV subtypes defined by necroptosis, R package WGCNA (Langfelder and Horvath, 2008) was used to convert gene expression data into gene co-expression networks. The samples were clustered based on the Pearson correlation value between each gene pair and average linkage, and the best β was selected by using the “pickSoftThreshold” function to satisfy the scale-free distribution, and the correlation coefficient was more than 0.85. Adjacency matrix was created for correlation strength description among the nodes, and was further transformed into topological overlap matrix (TOM). Next, hierarchical clustering tree was constructed by dynamic hybrid cutting technology to identify modules (parameters: height = 0.25, deepSplit = 2, minModuleSize = 80). After merging similar modules, the modules with strong correlation with OV subtypes defined by necroptosis were identified.

### Construction of necroptosis-related risk signature

The genes identified in the module were analyzed by univariate Cox regression, and the genes related to prognosis were included in the R packet “glmnet” for (LASSO Cox regression analysis. Then, the genes further screened by multivariate Cox regression analysis were used to construct risk models: NRRS = the sum of the multivariate LASSO regression coefficient of each gene × the normalized expression value of each gene transformed by log2 and z-score. To analyze the prediction effect of NRRS model on overall survival (OS), Kaplan-Meier survival curve and time-dependent receiver operating characteristic (tdROC) analyses were used.

### Enrichment analysis

The candidate gene set was obtained from the hallmark database, and the log_2_FC value of each gene was input into GSEA software for gene set enrichment analysis (GSEA) to explore the biological pathway of sample enrichment. P < 0.05 was considered to be significantly enriched after adjusting for Enrichment Score (ES). False discovery rate (FDR) < 0.05was defined as the cutoff value. The upregulation pathway was defined based on normalized enrichment scores (NES) > 0, and the downregulation pathway was defined based on NES <0.

### Construction of decision tree and nomogram

We used rpart package to build a decision tree based on age, stage, and grade and NRRS. Through the R package “rms,” a nomogram was generated. To evaluate the consistency between actual survival and the predicted results, calibration curves were plotted. The net benefit and clinical usefulness of the nomogram and NRRS model were determined by decision curve analysis (DCA) and tdROC.

### Statistical analysis

Statistical analysis was carried out by R 4.0.2 (https://www.r-project.org) to analyze the data and generate the results. R package “survminer” were performed to generate Kaplan-Meier curve, and “timeROC” were conducted to generate tdROC curve. The Wilcoxon rank-sum test were applied to compare continuous variables in two groups. Take *p* < 0.05 was the standard of statistical significance.

## Results

### Identification of necroptosis subtypes for OV

**FIGURE 1:**
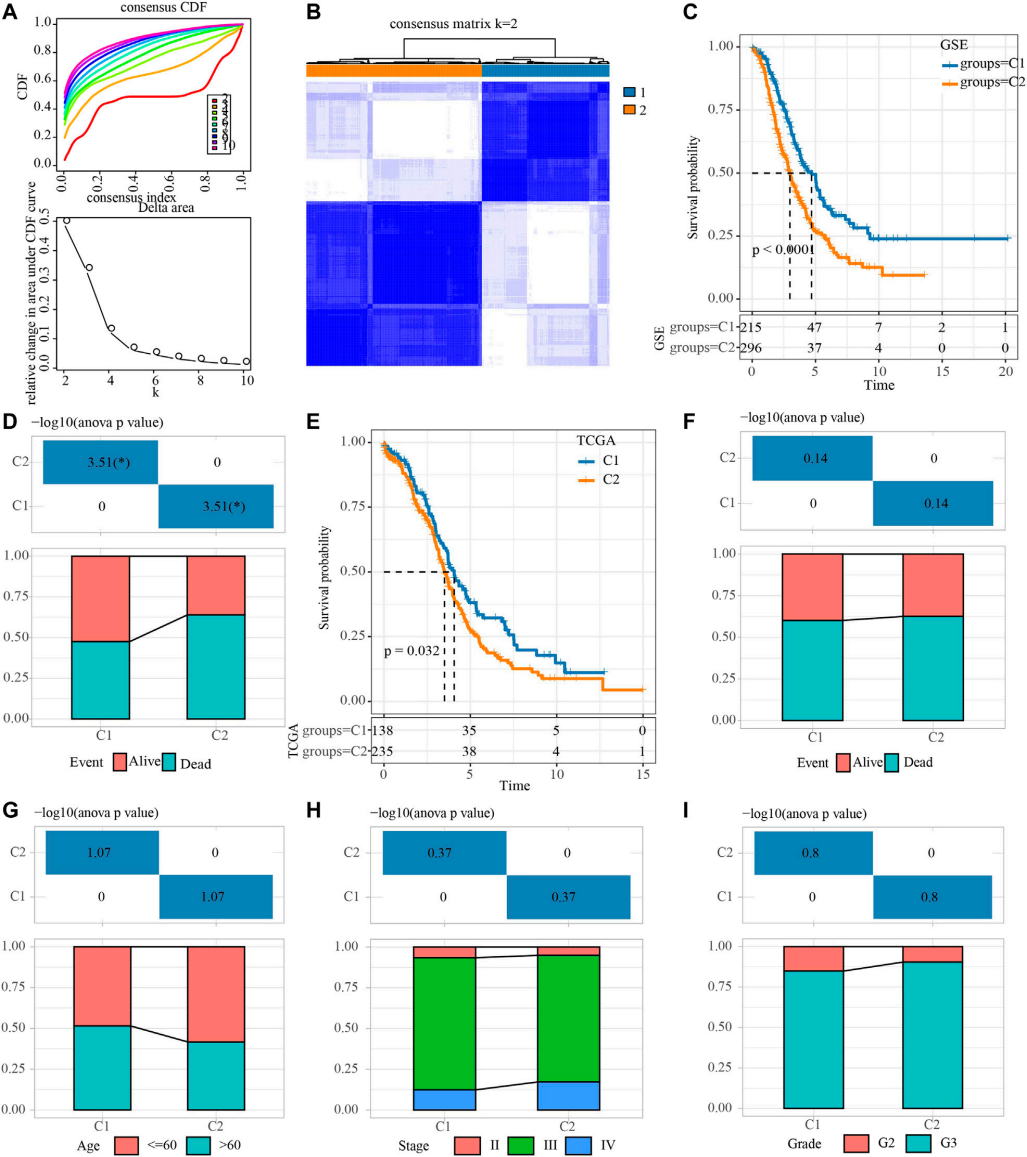
Identification of necroptosis subtypes for OV. **(A)** Consensus clustering heatmap for two subgroups. **(B)** CDF curve and the delta area of the clustering result. **(C)** Kaplan-Meier curve for LUAD patients in GSE dataset that merged four OV cohorts. **(D)** The distribution proportion of samples with different survival states in the two necroptosis subtypes of the GSE dataset that merged four OV cohorts. **(E)** Survival curve for LUAD patients in TCGA dataset. **(F)** Analysis of different survival states of two necroptosis subtypes in TCGA dataset. **(G–I)**: The characteristics of age, stage and grade of two necroptosis subtypes in TCGA dataset.

First of all, the sample expression data of four OV cohorts obtained from GEO were merged, and the deviation caused by batch effect was eliminated through the remove Batch Effect function of Limma package. Univariate Cox regression analysis of NRGs was carried out in the merged data, and 15 NRGs related to OV survival were identified. The merged OV samples were unsupervised consensus clustering. After comprehensive consideration of CDF curve and the delta area, KF2 was taken as the final number of clusters (Figures 1A,B). Consensus clustering was conducted in TCGA dataset (Supplementary Figure S1). Therefore, the two necroptosis subtypes that produce OV were cluster 1 (C1) and cluster 2 (C2). C1 was related to the better OV survival outcome of the GEO merger cohort (Figure 1C). And in the GSE with four cohorts, patients with survival accounted for more than 50% of C1, and patients with death accounted for a large proportion of C2 (Figure 1D). For OV samples in TCGA datasets, C1 was also associated with longer OS (Figure 1E). No statistical difference between the two subtypes in the proportion of patients with different survival states was found (Figure 1F). Although there was no significant difference in the proportion of age, stage, grade distribution between the two subtypes, it was obvious that there was a higher proportion of patients with age ≤60, stage Ⅳ and G3 in C2 (Figures 1G–I).

### Characterization of the genetic variation for two necroptosis subtypes

The mutation data downloaded from TCGA were analyzed in two necroptosis subtypes. 2,614 genes with mutation frequency >3 were first screened out. A total of 54 genes with significantly different mutation rates were identified by Fisher test between the two necroptosis subtypes. The first 20 genes with the most significant difference in mutation rate between the two necroptosis subtypes were shown in Supplementary Figure S2A. The overall SNV rate in C2 was higher than that in C1, and the mutation rate of CSMD3 in C2 was the highest, followed by MST1R, PRKDC, PLEKHG1, and SMG1. However, the mutation rate of SPTAN1, SRCAP, FAT1, ROBO1, UBR5 in C1 was significantly higher than that in C2. Tumor mutation burden, homologous recombination defect, fraction altered and number of segments did not show significant differences between the two subgroups (Supplementary Figure S2B).

### Necroptosis subtypes of OV showed different immune microenvironment and immunotherapeutic responses

**FIGURE 2:**
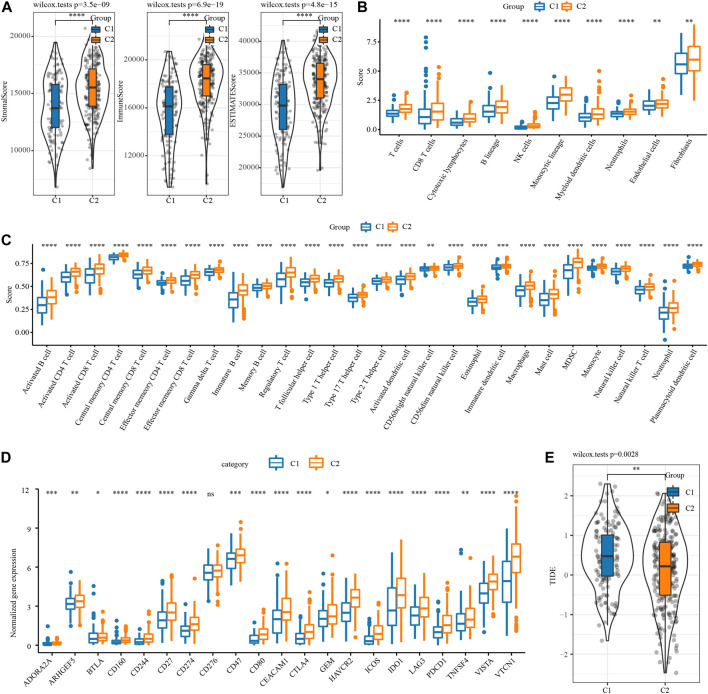
Immune microenvironment analysis and immunotherapy response prediction of necroptosis subtypes of OV. **(A)** The stromal score and immune score and tumor purity of two necroptosis subtypes in TCGA. **(B)** The infiltration scores of 10 immune cells calculated by MCP-Counter in the two necroptosis subtypes of OV. **(C)** The enrichment scores of 28 tumor-associated immune cells evaluated by ssGSEA in the two necroptosis subtypes of OV. **(D)** The box chart shows the association between the two necroptosis subtypes of OV and the expression of immune checkpoints. **(E)** TIDE score of two necroptosis subtypes of OV in TCGA. *p < 0.05, **p < 0.01, ***p < 0.001, ****p < 0.0001.

The difference of immune microenvironment between the two necroptosis subtypes was first evaluated by ESTIMATE. C2 showed significantly higher stromal score, immune score and the ESTIMATE score represented the overall microenvironment score relative to C1 (Figure 2A). Then, MCP-counter and ssGSEA were used to analyze the infiltration level of immune cells in the immune microenvironment between the two necroptosis subtypes. The infiltration score of 10 immune cells calculated by MCP-Counter in C2 was significantly higher than that in C1 (Figure 2B). 28 tumor-associated immune cells assessed by ssGSEA showed active enrichment in C2, which was significantly higher compared with C1 (Figure 2C). The results of Figures 2A–C reflected the abundant infiltration of immune cells in C2, and its anti-tumor immune microenvironment might be more active. However, C2 patients had the worst prognosis, which was not consistent with the immune characteristics of this subtype. One possible reason is that the anti-tumor response of C2 was blocked by simultaneously highly expressed immune checkpoints. To verify this conjecture, the expression of 21 immune checkpoint molecules from HisgAtlas database (Liu et al., 2017) was examined. It was found that except for CD276, the expression of 20 immune checkpoints in C2 was significantly up-regulated, such as CD274, CTLA4, GEM, IDO1, LAG3G, PDCD1 and so on (Figure 2D). Considering that the two necroptosis subtypes had different levels of immune checkpoint expression, the response of different necroptosis subtypes to immune checkpoint inhibitor (ICI) was predicted by TIDE algorithm. The TIDE score of C2 was significantly lower in both necroptosis subtypes, suggesting that C2 was more likely to respond to ICB treatment than C1 (Figure 2E).

### Identification of necroptosis subtype related gene modules

**FIGURE 3:**
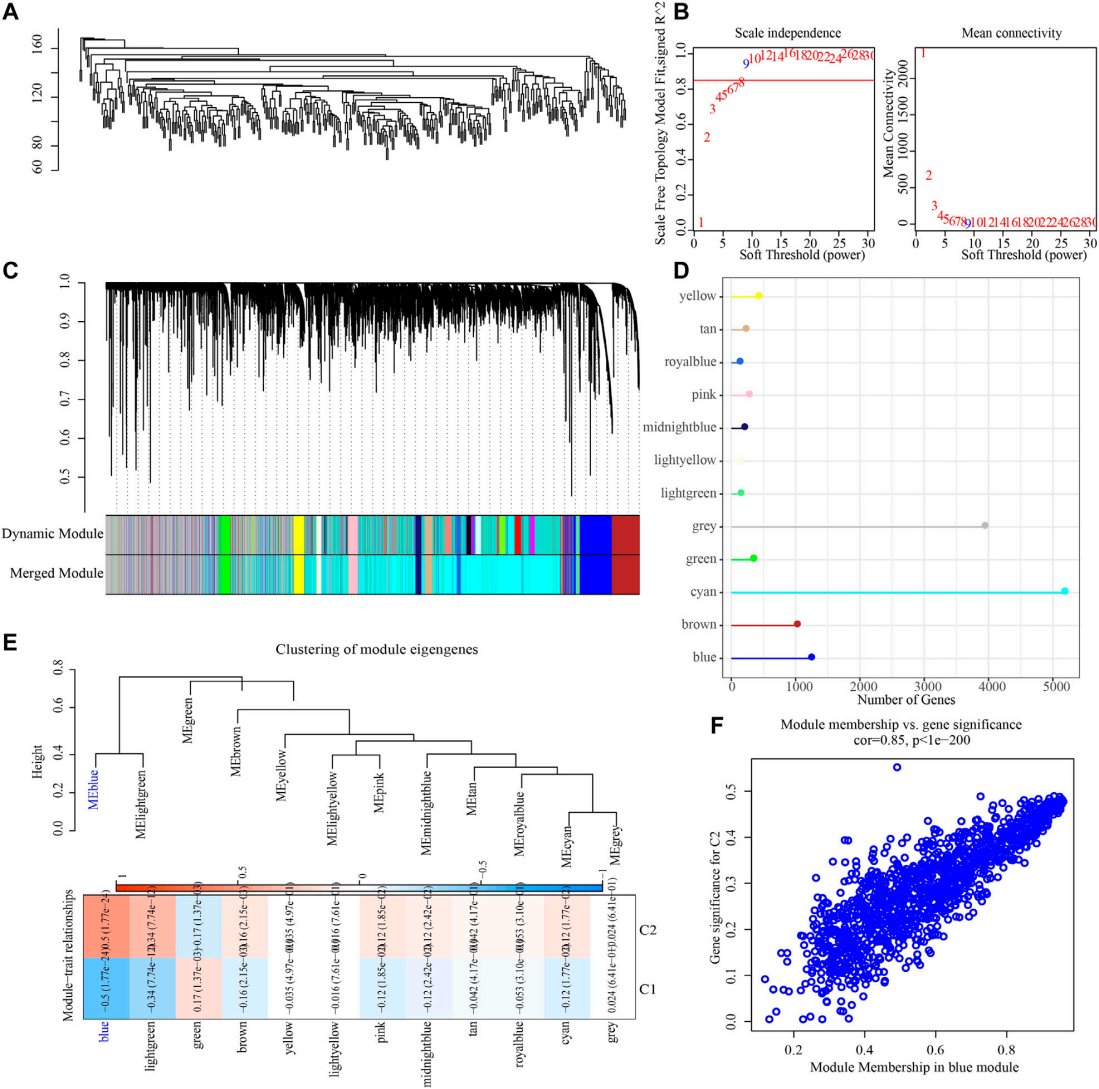
Identification of gene modules related to necroptosis subtypes. **(A)** The clustering tree of 373 OV samples in the TCGA dataset. **(B)** Analysis of scale-free exponent and average connectivity of various soft threshold powers. **(C)** Cluster dendrogram of the co-expression network modules. **(D)** The number of genes in each module. **(E)** The heatmap of the relationship between module eigengenes and necroptosis subtypes. **(F)** The association strength between gene significance (GS) and module membership (MM) for the C2 in the blue module.

To construct a co-expression network, WGCNA was used to cluster 373 OV samples from TCGA datasets (Figure 3A). When the lowest soft threshold power was 9, scale-free R2 >0.9, guaranteed a scale-free network (Figure 3B). A clustering tree diagram reflecting the relationship between different modules and clinical features was constructed by using adjacency matrix, and 12 modules were determined (Figures 3C, D). By looking for the correlation between feature genes and external features, we found that the blue module had the strongest correlation with the two necroptosis subtypes, significantly negative correlation with C1 and significant positive correlation with C2 (Figure 3E). The link between each gene and C2 in the blue module was also very high (Figure 3F).

### Construction of necroptosis-related risk score model based on hub gene in blue module

**FIGURE 4:**
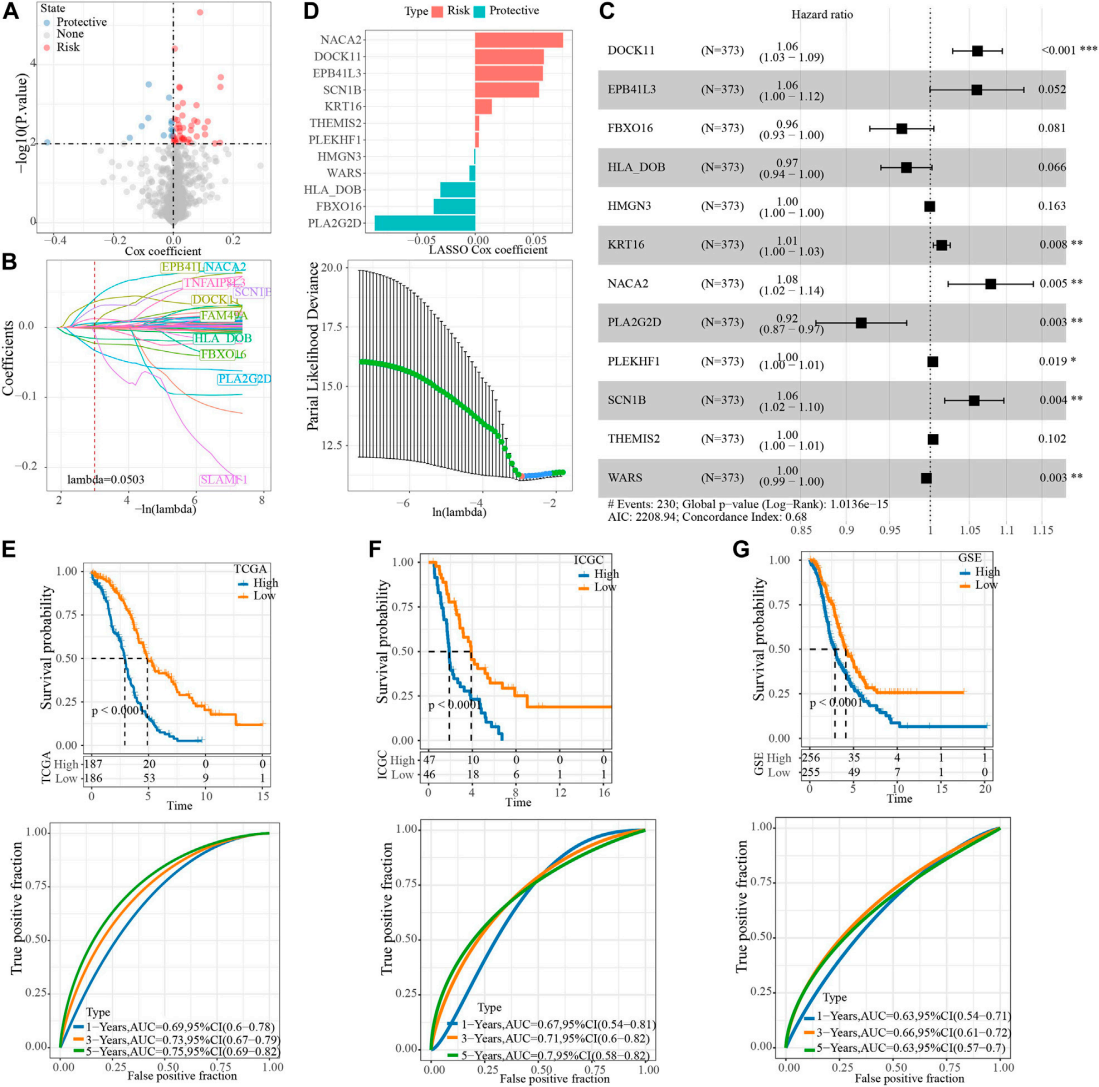
Construction of NRRS model based on hub gene in blue module. **(A)** The Cox coefficients of 55 genes related to OV survival. **(B)** LASSO Cox regression penalized the unimportant features in the regularization process. **(C)** The forest map shows the results of multivariate Cox regression analysis for 12 genes. **(D)** LASSO Cox coefficients of 12 genes. **(E)** Kaplan-Meier and tdROC curves of OS predicted by NRRS model in TCGA-OV cohort. **(F)** Kaplan-Meier and tdROC curves of OS predicted by NRRS model in ICGC dataset. **(G)** Kaplan-Meier and tdROC curves of OS predicted by NRRS model in GSE cohort.

To screen the hub genes in the blue module, the genes in the module were analyzed by univariate Cox regression analysis, and 55 genes related to OV survival were obtained. Among them, the higher expression level of 42 was associated with the higher death risk, and the higher expression of 13 was associated with the lower death risk (Figure 4A). LASSO Cox regression penalized the unimportant features in the regularization process, 24 genes were obtained, which need to be further analyzed (Figure 4B). Multivariate Cox regression analysis selected 12 of these genes to calculate the NRRS of the sample (Figure 4C). Among the 12 genes, NACA2, DOCK11, EPB41L3, SCN1B, KRT18, THEMIS2, PLEKHF1 were associated with poor OS of OV, while HMGN3, WAR3, HLA_DOB, FBXO16, PLA2G2D were associated with better OS (Figure 4D). Risk groups were divided based on the median of the sample NRRS in each cohort. The survival analysis was carried out between the high-risk and low-risk packets in each cohort, and the performance of the NRRS model in each queue was evaluated by tdROC curve. Among the 373 samples of TCGA, the survival rate of the high-risk group was significantly lower than that of the low-risk group in the long term and short term. TdROC curve showed that the NRRS model had better long-term predictive ability in the TCGA-OV cohort because its AUC for predicting 5-year OS was 0.75, it was higher than the AUCs for predicting 1-year (0.69) and 3-year (0.73) OS (Figure 4E). The high-risk group of OV samples obtained from ICGC was also associated with a worse prognosis outcome, with AUC of 0.67, 0.71, and 0.7 for 1 -, 3 -, and 5-year OS, respectively (Figure 4F). In the GSE cohort that merged four GEO datasets, the prognosis of the low-risk group was significantly better than that of the high-risk group. The model predicted 1 year AUC = 0.63, 3 years AUC = 0.66, and 5 years AUC = 0.63 of OS (Figure 4G).

### Single nucleotide variation and biological characteristics of necroptosis-related risk score model

**FIGURE 5:**
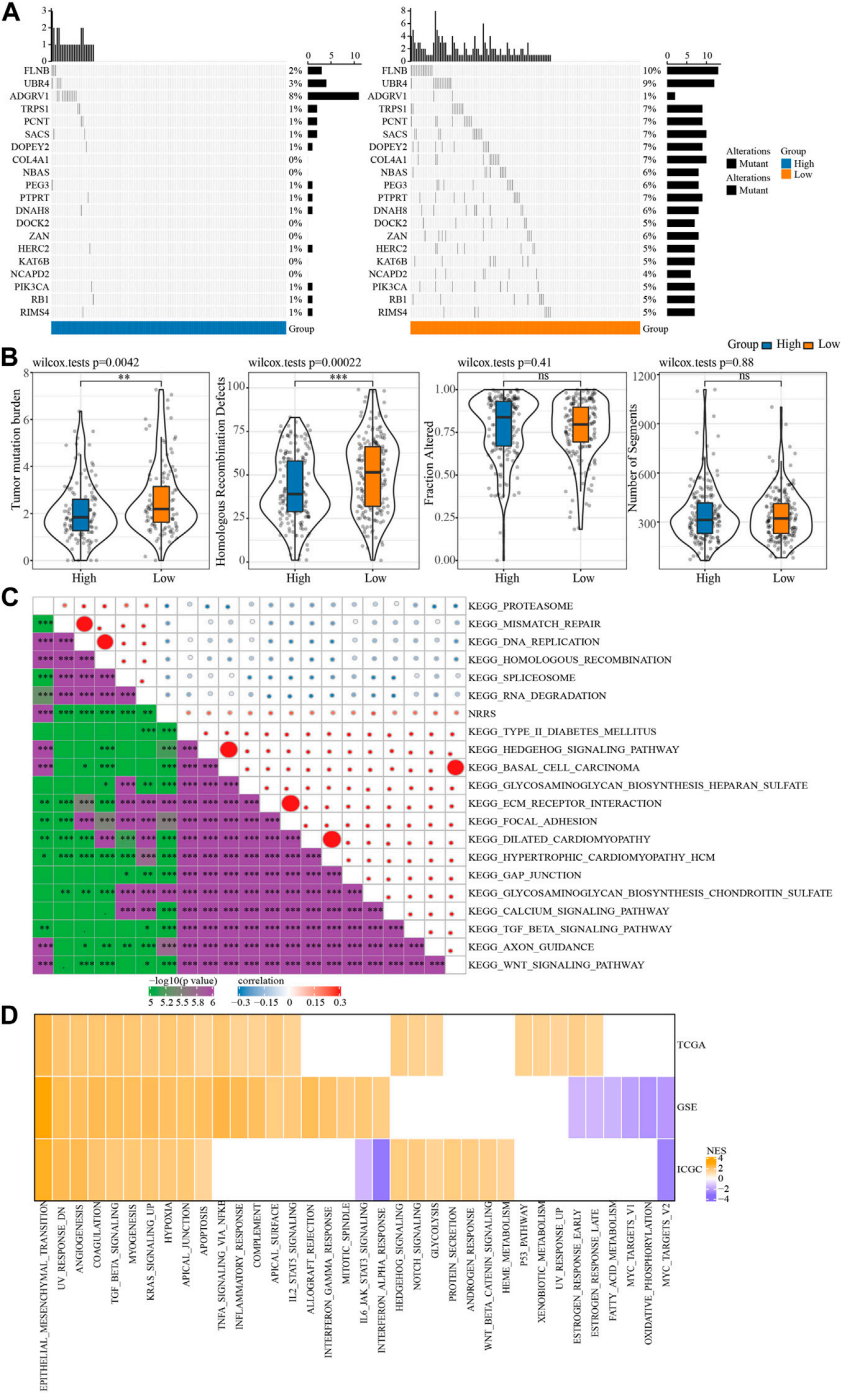
SNV and biological characteristics of NRRS model. **(A)** SNV in high-risk and low-risk groups. **(B)** TMB, homologous recombination defect, fraction altered, number of segments of high-risk group and low-risk group. **(C)** The correlation between NRRS and tumor biological pathway. **(D)** The high-risk group compared with the low-risk group in different pathways of NESs. *p < 0.05, **p < 0.01, ***p < 0.001, ****p < 0.0001.

We further explored the SNV and potential biological pathways related to NRRS. SNV existed in both high-risk and low-risk groups, and genetic mutations were more pronounced in the low-risk group than in the high-risk group, including but not limited to FLNB, UBR4, TRPS1, PCNT, SACS (Figure 5A). The TMB and homologous recombination defect characteristics of the low-risk group were significantly higher than those of the high-risk group (Figure 5B). The correlation between NRRS and tumor biological pathway was analyzed, and the results were shown in Figure 5C. Specifically, epithelial-mesenchymal transition, angiogenesis, coagulation, TGF beta signaling, myogenesis, KRAS signal up, hypoxia and apical junction and apoptosis were all up-regulated in the high NRRS group of the three datasets (Figure 5D).

### Construction of a decision tree model and a nomogram to improve the risk stratification of OS for OV patients

**FIGURE 6:**
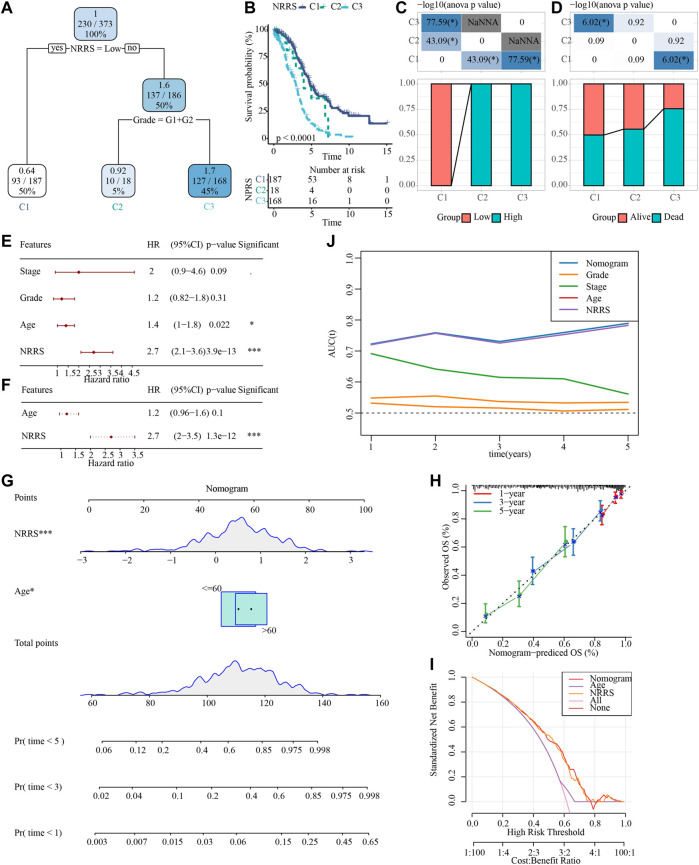
Construction of a decision tree model and a nomogram to improve the risk stratification of OS in OV patients. **(A)** Decision tree model based on NRRS and clinical factors (age, stage, grade). **(B)** Survival analysis of three risk subgroups of the decision tree. **(C)** The distribution of NRRS in three risk subgroups of the decision tree. **(D)** The survival status of patients in the three risk subgroups of the decision tree. **(E)** Univariate Cox regression analysis of NRRS and clinical factors of OV. **(F)** Multivariate Cox regression analysis of NRRS and age. **(G)** The nomogram constructed according to age and NRRS. **(H)** The calibration curve evaluates the proximity between the prediction line of the nomogram and the ideal 45-degree calibration line. **(I)** The decision curve shows the net income of NRRS and nomogram. **(J)** The tdROC curve displays the AUCs of NRRS and nomogram. *p < 0.05, ***p < 0.001.

To make NRRS more suitable for predicting the prognosis of OV, a decision tree model was constructed using the clinical factors (age, stage, grade) of OV in TCGA and NRRS, and three different clusters:M1, M2 and M3 were established (Figure 6A). There were significant differences in OS among the three clusters (Figure 6B). All samples in M1 belonged to low NRRS group, while M2 and M3 belonged to high NRRS group (Figure 6C). The proportion of surviving patients in M1 was the highest among the three clusters, followed by M2, and finally M3 (Figure 6D). To construct a nomogram, univariate Cox regression analysis was carried out first, and the age and NRRS fits very well (Figure 6E). Multivariate Cox regression showed that NRRS was an independent prognostic variable for OV (Figure 6F). A nomogram was constructed according to age and NRRS (Figure 6G). The calibration curve showed that the prediction line of nomogram was close to the ideal 45° calibration line, indicating that nomogram had a certain degree of accuracy (Figure 6H). The decision curve showed that NRRS and nomogram have the highest net income (Figure 6I). And tdROC curve displayed that the AUC of NRRS and nomogram was very similar, both above 0.7 (Figure 6J).

## Discussion

OV is a complex disease with multiple subtypes, each of which has different histopathology and different responses to treatment. Accurate classification and typing of OV can reliably predict disease progression and provide insight into the potentially targeted molecular mechanisms unique to each subtype (Cook and Vanderhyden, 2019). The study of Seehawer et al. provides a revolutionary insight that necroptotic microenvironment direct the lineage commitment of liver cancer and thus determine cancer subtypes (Seehawer et al., 2018). It is unclear whether necroptosis can affect the subtypes of other cancers. In recent years, several studies have focused on the effects of necroptosis-related genes on cancer typing, prognosis and biological effects (He et al., 2022; Nie et al., 2022; Xin et al., 2022). In this study, we systematically studied the effects of necroptosis on OV typing, prognosis, TMB, tumor microenvironment, immunotherapy response and biological pathway by bioinformatics analysis, which might be provide new molecular insights for necroptosis in OV.

First of all, we classified OV into two necroptosis subtypes according to 15 OV prognosis-related NRGs out of 74 NRGs. Compared with C1, C2 with a worse prognosis. The possible reason was that C2 showed a high immunosuppressive microenvironment. In OV, a large number of immunosuppressive cells, including tumor-associated macrophages, regulatory T cells (Tregs), myeloid-derived suppressor cells (MDSCs) and Tumor associated dendritic cells, act as accomplices to coordinate highly complex immunosuppressive networks, inhibit anti-tumor immunity and help tumor cells escape immune attacks (Cai and Jin, 2017). Besides, in the early stage of tumor, the adaptive immune resistance mechanism may occur in patients with high CD8T cell density and predict a poor prognosis of the tumor (Peske et al., 2015). In the immunosuppressive environment, it is rich in immunosuppressive molecules such as IDO, PD-1, PD-L1, VISTA, LAG3, etc (Dempke et al., 2017; Drakes and Stiff, 2018). In C2, the levels of above immune cells and immunosuppressive molecules were higher than those in C1, which explained why the prognosis of C2 iwas worse than that of C1.

Then, the blue module most related to necroptosis subtype was identified by constructing a co-expression network, and the hub gene of the module was identified by LASSO Cox regression analysis. A NRRS model containing 12 genes (NACA2, DOCK11, EPB41L3, SCN1B, KRT18, THEMIS2, PLEKHF1, HMGN3, WAR3, HLA_DOB, FBXO16, PLA2G2D) was constructed. Among all 12 prognostic related genes, 8 genes (EPB41L3, SCN1B, KRT18, THEMIS2, PLEKHF1, HLA_DOB, FBXO16, PLA2G2D) (Dafou et al., 2010; Trisdale et al., 2016; Li et al., 2020; Brummelhuis et al., 2021; Ji et al., 2021; Zheng et al., 2021; Huang et al., 2022; Zhao et al., 2022) have been reported to be involved in tumorigenesis of OC or to be important predictors of overall survival. This implies that our bioinformatics analyses using cohorts have prognostic value. The remaining 4 genes have not previously been found to be associated with the prognosis of ovarian cancer and may serve as new potential biomarkers for the disease.

NRRS had many far-reaching clinical significances. First, it was related to the genomic stability of tumors. The NRRS low-risk group showed higher levels of SNV, TMB and homologous recombination defect. Second, it was related to the biological process of tumor. Specifically, compared with the NRRS low-risk group, epithelial-mesenchymal transition, angiogenesis, coagulation, TGF beta signaling, myogenesis, KRAS signal up, hypoxia and apical junction and apoptosis pathways were significantly up-regulated in the high-risk group. Third, NRRS was an independent prognostic variable of OV, and it was more accurate than other clinical parameters in predicting the prognosis of OV. And the decision tree and nomogram combined with NRRS and other clinical factors improved the risk stratification of OS in patients with OV.

However, some limitations of this study must be recognized. This study was purely from the bioinformatics analysis of the public database, the sample size of each cohort was relatively small, clinical information was prone to deviation, large-scale, multicenter, prospective studies are needed to further confirm our results. And the impact of the model needs biological experiments and clinical data to support it. In addition, the specific molecular mechanism of the model in OV remains to be further explored.

To sum up, OV was divided into two necroptosis subtypes in this study. There were significant differences in OS, immune cell infiltration, immune checkpoint expression and applicability to immunotherapy between patients with different subtypes. Moreover, a NRRS model was constructed to identify high-risk patients with OV, and combined with clinical factors to build a decision tree and nomogram to optimize the risk stratification of OS. Our research may provide molecular insights into the effects of necroptosis in cancer.

## Data Availability

The datasets presented in this study can be found in online repositories. The names of the repository/repositories and accession number(s) can be found in the article/Supplementary Material.
