# Supplementary material for: Identification of necroptosis subtypes and development of necroptosis-related risk score model for in ovarian cancer
Source: Front Genet. 2022 Dec 8;13:1043870. doi: 10.3389/fgene.2022.1043870 (PMC9773578; doi:10.3389/fgene.2022.1043870)
Supplement: Supplementary file 1 [file Table1.DOCX]

**Description of supplementary figures**

**Figure S1 Consensus clustering in TCGA dataset**

**Figure S2 Characterization of genetic variation of two necroptosis subtypes**

A: The waterfall map shows the first 20 genes with the most significant difference in mutation rates between the two necroptosis subtypes. B: The violin picture shows the relation between tumor mutation burden, homologous recombination defect, fraction altered, number of segments and necroptosis subgroups, respectively.
