# Supplementary figures and images for: Identification of necroptosis subtypes and development of necroptosis-related risk score model for in ovarian cancer
Source: Front Genet. 2022 Dec 8;13:1043870. doi: 10.3389/fgene.2022.1043870 (PMC9773578; doi:10.3389/fgene.2022.1043870)

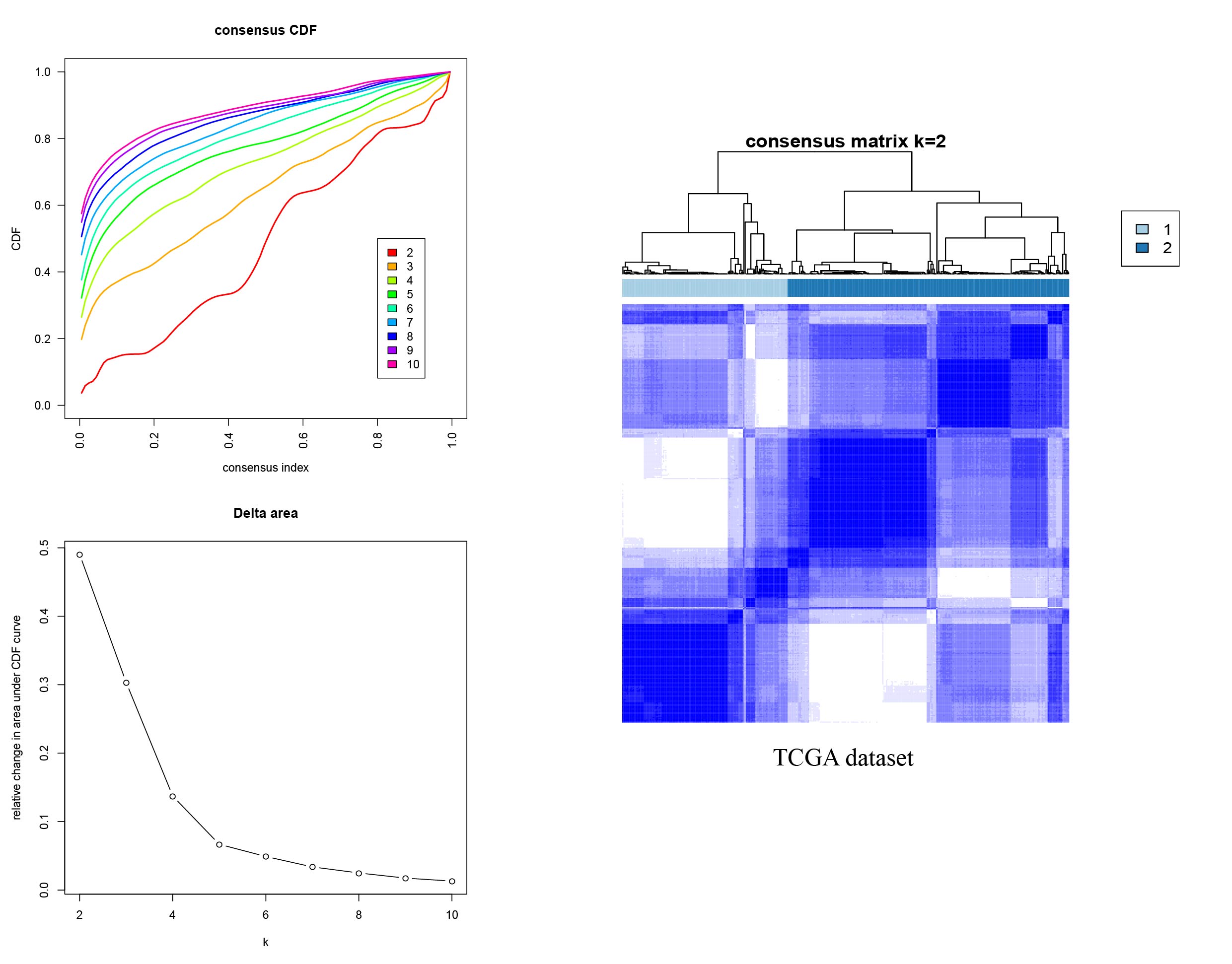

Supplement: Supplementary file 2 [file Image1.JPEG]

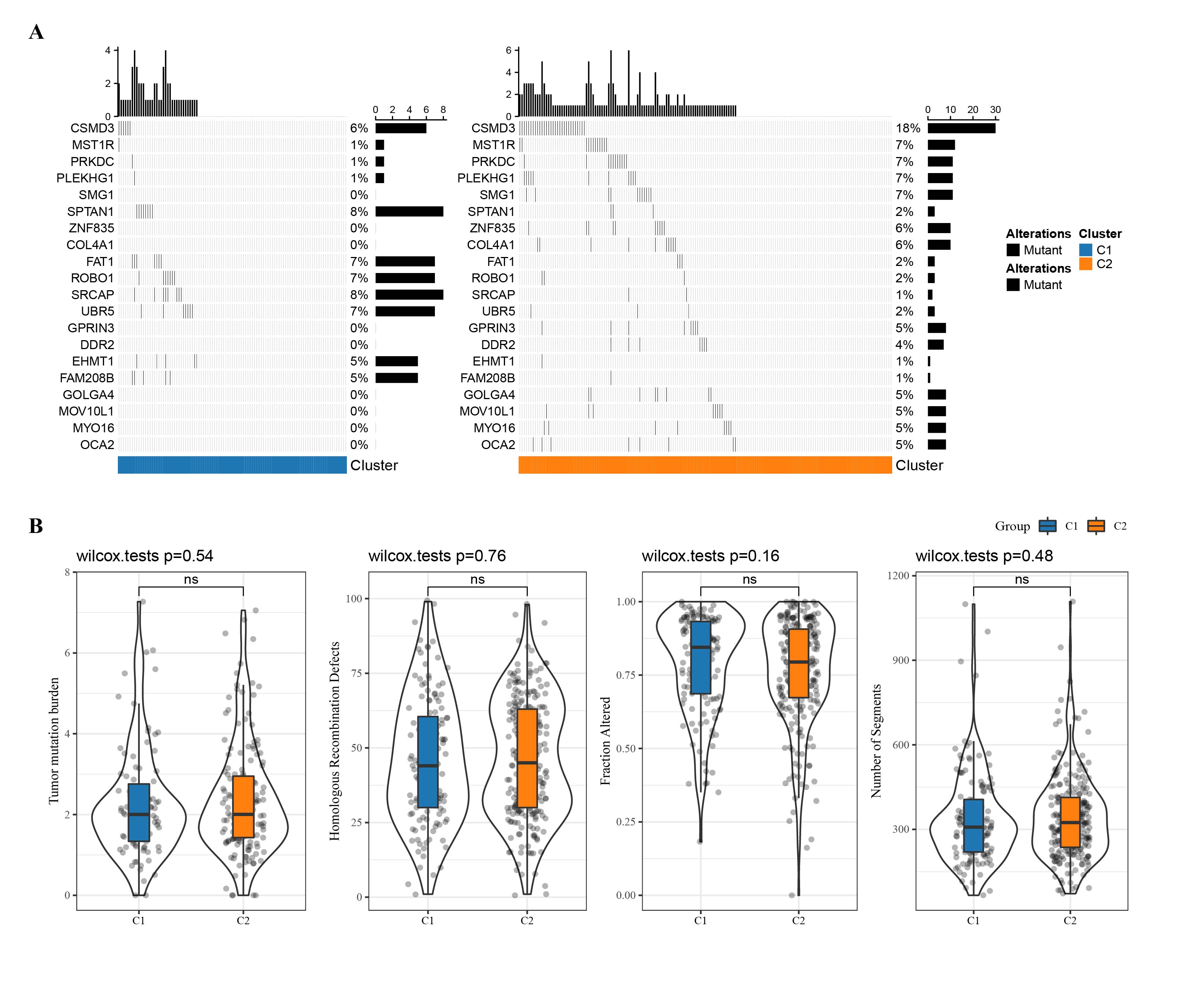

Supplement: Supplementary file 3 [file Image2.JPEG]
