# Supplementary material for: Identification of necroptosis subtypes and development of necroptosis-related risk score model for in ovarian cancer
Source: Front Genet. 2022 Dec 8;13:1043870. doi: 10.3389/fgene.2022.1043870 (PMC9773578; doi:10.3389/fgene.2022.1043870)
Supplement: Supplementary file 4 [file Table2.docx]

For the data analyzed in this study please see:

https://www.jianguoyun.com/p/DWHn63EQ69f3Chj_6tYEIAA
